# Comparative Study on the MDR Reversal Effects of Selected Chalcones

**DOI:** 10.1155/2011/530780

**Published:** 2010-12-29

**Authors:** A. B. Ivanova, D. I. Batovska, I. T. Todorova, B. A. Stamboliyska, J. Serly, J. Molnar

**Affiliations:** ^1^Institute of Organic Chemistry with Centre of Phytochemistry, Bulgarian Academy of Sciences, Acad. G. Bonchev Street Bl. 9, Sofia 1113, Bulgaria; ^2^Department of Medical Microbiology and Immunobiology, Faculty of Medicine, University of Szeged, 10 Dóm tér, 6720 Szeged, Hungary

## Abstract

Based on the structure of three previously established lead compounds, fifteen selected chalcones were synthesized and evaluated for their multidrug resistance (MDR) reversal activity on mouse lymphoma cells. The most active chalcones were stronger revertants than the positive control, verapamil. In the model of combination chemotherapy, the interactions between the anticancer drug doxorubicin and two of the most effective compounds were measured *in vitro*, on human MDR1 gene transfected mouse lymphoma cells, showing that the type of interaction for one of these compounds was indifferent while that for the other one was additive. Furthermore, two chalcones inhibited 50% of cell proliferation in concentration of around 0.4 *μg*/mL and were from 2- to 100-fold more active than the most chalcones. The structure-activity relationships were obtained and discussed in view of their usefulness for the design of chalcone-like P-gp modulators and drugs able to treat resistant cancers.

## 1. Introduction

Multidrug resistance (MDR) against anticancer drugs is one of the major obstacles to successful chemotherapy. It refers to the ability of tumors to develop resistance to a number of structurally and functionally unrelated chemotherapeutic agents [1]. In malignant cells, MDR is predominantly mediated by the overexpression of P-glycoprotein (P-gp), which is a member of the adenosine triphosphate (ATP)-binding cassette (ABC) superfamily. P-gp recognizes a wide range of compounds including anticancer drugs and actively transports them out of the cells thereby lowering their intracellular concentrations and pharmacological effects [2]. For this reason, developing inhibitors of P-gp-mediated drug efflux is of potential clinical value.

Up to now a great variety of P-gp inhibitors has been discovered. Amongst them, flavonoids constitute the third generation, a nonpharmaceutical category of revertants. The effects produced by some of these components are found to be comparable to those of the well-known P-gp inhibitors verapamil and cyclosporine [3]. Within the last decade, interest intensified in the flavonoid subclass of chalcones. Chalcones (1,3-diarylprop-2-en-1-ones) are open-chain flavonoids consisting of two aromatic rings (A and B) that are joined by a three-carbon *α*,*β*-unsaturated carbonyl system. They display a wide pharmacological spectrum including the dual effects of anticancer and MDR reversal activities, which makes them promising agents for cancer chemotherapy [4]. Chalcones are supposed to overlap two binding sites of the nucleotide-binding domain (NBD2) of P-gp [5]. The ring A and the unsaturated carbonyl system are likely to bind to the ATP-site while the ring B probably binds to the steroid-interaction region. Some studies of variously substituted chalcones pointed at particular structural features related to the MDR reversal activity. Thus, the presence of a hydrophobic *p*-substituent in ring B, such as a halogen or an alkoxy group was considered favourable [5, 6]. A basic group was found efficient, when being present on only one of the aromatic rings of the chalcone template. However, this feature was not always sufficient for exhibiting P-gp inhibitory activity [7]. Also, *m*-dimethoxy motif on chalcone framework was found crucial, presumably because in this case electron donor atoms with optimal spatial distances were introduced.

In our previous study, we investigated a set of chalcones with 3′,4′,5′-trimethoxylated ring A and a variously substituted ring B [8]. Amongst them, the compounds with 4-chloro (**1**), 4-dimethylamino (**2**), and 4-methoxy (**3**) groups were the most promising modulators of P-gp-efflux pump, all of them being much stronger inhibitors than verapamil. Based on these lead compounds, in the present study we synthesized a series of 4-chloro-, 4-methoxy-, and 4-dimethylaminochalcones with a variously methoxylated ring A and examined their MDR reversal activity on human *MDR1* gene-transfected mouse lymphoma cells (L 5178 Y). The observed effects were compared with those of the lead compounds (**1**–**3**). Structure-activity relationships were discussed in view of their usefulness for the design and synthesis of chalcone-like MDR revertants.

## 2. Experimental

### 2.1. Chemistry

#### 2.1.1. General

Infrared and UV spectra were recorded on Bruker IFS 113V and Helios gamma UV-vis spectrophotometers, respectively. ^1^H and ^13^C NMR spectra were obtained with Bruker AM 250 spectrometer with tetramethylsilane as internal reference. Chemical shifts are given in ppm (*δ*-scale); coupling constants (*J*) are in Hz. Splitting patterns are described as singlet (s), doublet (d), triplet (t), and multiplet (m). Mass spectral analyses were accomplished on a Hewlett-Packard 5972 using Mass Selective Detector with EI (70 eV). The melting points were obtained using Mel-Temp 1102D-230 VAC and were uncorrected. The reactions were monitored on silica gel 60 F254 using PE/acetone 7 : 3.

#### 2.1.2. Synthesis and Spectral Data of Chalcones **10** and **16**

3,4-Dimethoxyacetophenone (150 mg, 0.8 mmol) or 2,5-dimethoxyacetophenone (132 *μ*L, 0.7 mmol) was added to equimolar quantities of *p*-chlorobenzaldehyde and dissolved in MeOH (0.8 mL). To this solution 6 M NaOH (0.6 mL) was added and the reaction mixture was stirred for 30 min and then kept in refrigerator overnight. The product crystals were filtrated and washed with ice water and cold MeOH to neutral reaction. The resulting chalcones were purified by recrystallization from MeOH.

(*E*)-3-(4-Chlorophenyl)-1-(3,4-dimethoxyphenyl)prop-2-en-1-one (**10**): Yield (212 mg, 84%), white crystals, m.p. 110–113°C. Anal. Calcd. for C_17_H_15_ClO_3_: C, 67.44; H, 4.99; Cl, 11.71. Found: C, 67.49; H, 5.04; Cl, 11.78; UV (MeOH, nm): 228, 313; IR (KBr, cm^−1^): 1655 (C=O), 1560 (C=C), 1325 (C–O–Ar), 810 (C–Cl); ^1^H NMR (250 MHz, CDCl_3_, *δ*/ppm) 7.75 (d, *J* = 15.5 Hz, 1H, H-*β*), 7.67 (dd, *J*_1_ = 8.5 Hz, *J*_2_ = 2.0 Hz, 1H, H-6′), 7.62 (d, *J* = 2.0 Hz, 1H, H-2′), 7.56–7.59 (m, 2H, H-3, H-5), 7.52 (d, *J* = 15.5 Hz, 1H, H-*α*), 7.37–7.40 (m, 2H, H-2, H-6), 6.93 (d, *J* = 8.5 Hz, 1H, H-5′), 3.97 (s, 6H, 2 × OCH_3_). ^13^C NMR (250 MHz, CDCl_3_, *δ*/ppm) 188.2 (C=O), 153.4 (C-4′), 149.3 (C-3′), 142.4 (C-*β*), 136.2 (C-4), 133.6 (C-1), 131.2 (C-1′), 129.5 (C-3, C-5), 129.2 (C-2, C-6), 123.0 (C-6), 122.1 (C-*α*), 110.8 (C-5), 110.0 (C-2), 56.1 (2 × OCH_3_); MS EI (m/z, %) 302 (M^+·^, 100).

(*E*)-3-(4-Chlorophenyl)-1-(2,5-dimethoxyphenyl)prop-2-en-1-one (**16**): Yield (219 mg, 87%), yellow crystals, m.p. 74–76°C. Anal. Calcs. for C_17_H_15_ClO_3_: C, 67.44; H, 4.99; Cl, 11.71. Found: C, 67.51; H, 5.02; Cl, 11.79; UV (MeOH, nm): 227, 309; IR (KBr, cm^−1^): 1660 (C=O), 1560 (C=C), 1328 (C–O–Ar), 816 (C–Cl); ^1^H NMR (250 MHz, CDCl_3_, *δ*/ppm) 7.62 (d, *J* = 15.8 Hz, 1H, H-*β*), 7.52–7.58 (m, 2H, H-3, H-5), 7.43 (d, *J* = 15.8 Hz, 1H, H-*α*), 7.37–7.39 (m, 2H, H-2, H-6), 7.23 (d, *J* = 3.0 Hz, 1H, H-6′), 7.07 (dd, *J*_1_ = 9.0 Hz, *J*_2_ = 3.0 Hz, 1H, H-4′), 6.97 (d, *J* = 9.0 Hz, 1H, H-3′), 3.89 (s, 3H, OCH_3_), 3.84 (s, 3H, OCH_3_); ^13^C NMR (250 MHz, CDCl_3_, *δ*/ppm) 192.0 (C=O), 153.7 (C-2′), 152.7 (C-5′), 141.5 (C-*β*), 136.1 (C-4), 133.7 (C-1), 129.5 (C-3, C-5), 129.1 (C-2, C-6), 127.3 (C-1′, C-*α*), 119.4 (C-4′), 114.5 (C-3′), 113.4 (C-6′), 56.5 (OCH_3_), 55.9 (OCH_3_); MS EI (m/z, %) 302 (M^+·^, 100).

### 2.2. Biological Assays

#### 2.2.1. Cell Cultures

L5178 mouse T-cell lymphoma cells (ECACC cat. no. 87111908, U.S. FDA, USA) were transfected with pHa MDR1/A retrovirus [16]. The *MDR1*-expressing cell line was selected by culturing the infected cells with 60 ng/mL colchicine to maintain the expression of the MDR phenotype. Parental mouse T-cell lymphoma cells and the human *MDR1*-transfected subline were cultured at 37°C in McCoy's 5A medium supplemented with 10% heat-inactivated horse serum, L-glutamine, and antibiotics. The mouse lymphoma cell line was maintained in a 5% CO_2_ atmosphere at 37°C.

#### 2.2.2. Assay for Reversal of MDR in Tumor Cells

The cells were adjusted to a density of 2 × 10^6^ cell/mL, resuspended in serum-free McCoy's 5A medium and distributed in 0.5 mL aliquots into Eppendorf centrifuge tubes. The tested compounds were added at different final concentrations (4.0 and 40.0 *μ*g/mL), and the samples were incubated for 10 min at room temperature. Ten *μ*L (5.2 *μ*M final concentration) of the indicator rhodamine 123 (Sigma, St Louis, MO, USA) was added to the samples and the cells were incubated for a further 20 min at 37°C, washed twice and resuspended in 0.5 mL phosphate-buffered saline (PBS) for analysis. The fluorescence of the cell population was measured with a FACS Star Plus flow cytometer (Beckton, Dickinson and Company, Franklin Lakes, NJ, USA). Verapamil (EGIS Pharmaceuticals PLC, Budapest, Hungary) was used as a positive control in the rhodamine 123 exclusion experiments. The percentage mean fluorescence intensity was calculated for the treated MDR and parental cell lines as compared with the untreated cells. The fluorescence activity ratio (FAR) was calculated *via* the following equation, on the basis of the measured fluorescence values:

(1) $$\text{FAR}=\frac{\text{MDR}\;\;\text{treated}/\text{MDR}\;\;\text{control}}{\text{parental}\;\;\text{treated}/\text{parental}\;\;\text{control}}.$$

#### 2.2.3. Assay for Antiproliferative Effect

The effects of increasing concentrations of the drugs alone and their combinations with resistance modifiers on cell growth were tested in 96-well flat-bottomed microtitre plates. The compounds were diluted in two steps in a volume of 50 *μ*L medium to the final concentration of 25 *μ*g/mL. A total of 6 × 10^3^ cells in 50 *μ*L of medium were then added to each well, with the exception of the medium control wells. The culture plates were further incubated at 37°C for 72 h; at the end of the incubation period, 20 *μ*L of MTT solution (thiazolyl blue solved in PBS to a final concentration of 5 mg/mL) were added to each well. After further incubation at 37°C for 4 h, 100 *μ*L of sodium dodecyl sulphate (SDS) solution (10%) were measured into each well and the plates were further incubated at 37°C overnight. The cell growth was determined by measuring the optical density (OD) at 550 nm (ref. 630 nm) with a Multiscan EX ELISA reader (Thermo Labsystems, Cheshire, WA, USA). Inhibition of cell growth was determined as a percentage according to the formula:

(2) $$100-\left[\frac{\text{OD}\;\text{treated}\;\text{cells}-\text{OD}\;\text{medium}\;\;\text{control}}{\text{OD}\;\text{cell}\;\text{control}-\text{OD}\;\text{medium}\;\text{control}}\times 100\right].$$

### 2.3. Checkerboard Microplate Method

The microplate method was applied to study the effects of drug interactions between the resistance modifiers **10** and **14** and the anticancer agent doxorubicin on cancer cells as an *in vitro *model of combination chemotherapy. The dilutions of doxorubicin (A) were made in a horizontal direction and the dilution of the resistance modifiers (B) vertically in the microtitre plate, in a volume of 100 *μ*L. The cell suspension in the tissue culture medium was distributed into each well in 50 *μ*L containing 1 × 10^4^ cells. The plates were incubated in a CO_2_-incubator at 37°C for 48 h. The cell growth rate was determined after MTT staining and the intensity of the blue colour was measured on a Multiscan EX ELISA reader. Drug interactions were evaluated according to the system in Table 3.

### 2.4. Computational Details

The quantum chemical calculations were performed using the GAUSSIAN-98 program package [17]. The geometries of all possible conformational isomers of studied chalcones were fully optimized at B3LYP/6-31G∗ level of theory without constraints.

The log *P* values of chalcones were calculated online by Marvin calculator [18].

The correlation coefficients (*r*) and their significance were computed by Microsoft Office Excel software.

## 3. Results and Discussion

### 3.1. Chemistry

The fifteen chalcones (**4**–**18**) selected for this study were synthesized by Claisen-Schmidt condensation between 2,3,4-trimethoxy-, 2,4,6-trimethoxy-, 2,4-dimethoxy-, 3,4-dimethoxy-, and 2,5-dimethoxyacetophenone and three aryl aldehydes substituted at *p*-position with chloro-, methoxy-, or dimethylamino groups (Table 1). The desired products were obtained in high purity and in yields over 80%, following procedures described previously [19]. Their structures were established with UV, IR, NMR, mass spectrometry and elemental analysis. ^1^H NMR spectra showed that only transchalcones were obtained. Despite the great number of synthetic chalcones reported in the literature no data were found about the relatively simple compounds **10** and **16**. Their synthesis and spectral characterization were described in Section 2.1. The rest of chalcones have been previously reported by other authors as this is indicated in Table 1.

### 3.2. MDR Reversal Activity Evaluation

**Figure 1:**
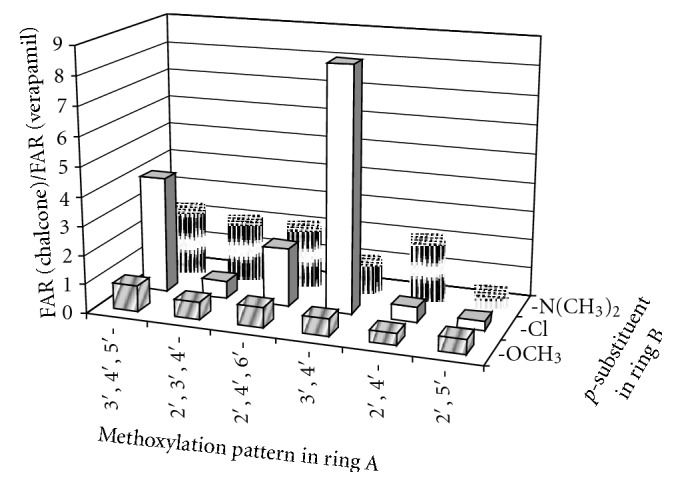
Influence of the substituent pattern in rings A and B on the MDR reversal of chalcones **1**–**18** on mouse lymphoma cell line L 5178 T.

The MDR reversal effects of the synthesized chalcones were examined on human *MDR1* gene-transfected mouse lymphoma cells (L 5178 Y) in concentrations of 4.0 and 40.0 *μ*g/mL by flow cytometry following the procedure employed by us for the lead compounds (Table 2) [8]. The well-known MDR modifier verapamil was used as a positive control. To compare the activities of chalcones (**4**–**18**) examined in this study with those of the previously tested compounds (**1**–**3**), we applied the ratios between the chalcones' activity at 4.0 *μ*g/mL and the activity shown in the same experiment by the positive control verapamil, that is, FAR (chalcone)/FAR (verapamil) (Figure 1).

**Figure 2:**
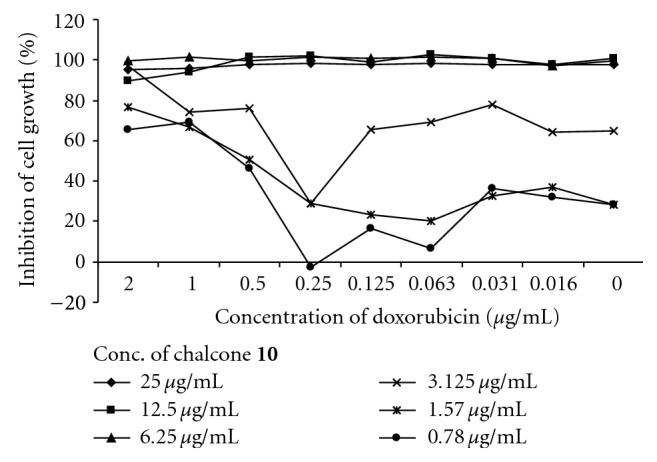
Evaluation of antiproliferative effect of chalcone **10 **in combination with doxorubicin.

**Figure 3:**
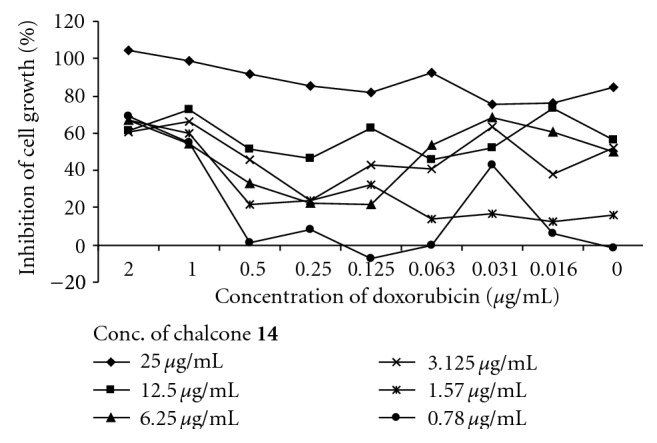
Evaluation of antiproliferative effect of chalcone **14 **in combination with doxorubicin.

Two peaks were observed in the histograms of fluorescence for the most chalcones suggesting that these compounds did not inhibit specifically the MDR efflux. This phenomenon may be due to a cell sensitive effect or due to a simultaneous action of another bioactivity. Despite this fact, the majority of compounds showed effects, which were a bit lesser than or comparable with that of verapamil. The most active chalcones **5**, **7**, **8**, **10**, **11**, and** 14** were stronger revertants than the positive control. However, the inhibition of compound **10** was slightly dose dependent because both of the two concentrations were at the saturation zone of biological effect. At the lower concentrations of 0.4 and 0.04 *μ*g/mL, chalcone **10** showed more distinct MDR activity with fluorescent activity ratio (FAR) values of 56.1 and 11.9, respectively. The reversal effects of two of the most active compounds **10** and **14** were measured *in vitro* in combination with the anticancer drug doxorubicin (Figures 2 and 3). The interaction between **10** and doxorubicin (FIX = 1.78) was found to be indifferent while **14** (FIX = 0.99) had an additive effect.

The antiproliferative effects of chalcones **4**–**18** were examined against the human MDR1 gene-transfected mouse lymphoma cells by MTT test (Table 4). Two of compounds, **5** and **17**, inhibited 50% of cell proliferation in concentrations of around 0.4 *μ*g/mL and were from 2- to 100-fold more active than the most chalcones.

P-gp inhibitory activity of chalcones and structurally similar compounds such as flavones and propafenones correlates with hydrophobicity, H-bond acceptor strength, the spatial arrangement of H-bond acceptors, and molar refractivity [8, 20, 21]. Influence of these and other molecular characteristics on the MDR reversal activity of chalcones **1**–**18** are discussed below.

### 3.3. Influence of Hydrophobicity of Chalcones on Their MDR Reversal Activity

The log *P* value, which is a measure of hydrophobicity, was calculated for chalcones **1**–**18** and was found to be higher than 2.9 for all of them (Table 1). Thus these compounds met one of the requirements for highly effective P-gp modulators [22]. A moderate but statistically significant positive correlation (*r* = 0.4, *P* < .05, *n* = 18) was found between the log *P* values and the MDR reversal effects within the entire set of chalcones. However, only the half of the six most hydrophobic chalcones **1**, **7**, and **10** (log *P* > 4.0) showed high activity (Table 1, Figure 1). This fact suggests that hydrophobicity is an important but not sufficient parameter for chalcones to inhibit P-gp/MDR1.

### 3.4. Influence of the Substitution Pattern of Chalcones on Their MDR Reversal Activity

**Figure 4:**
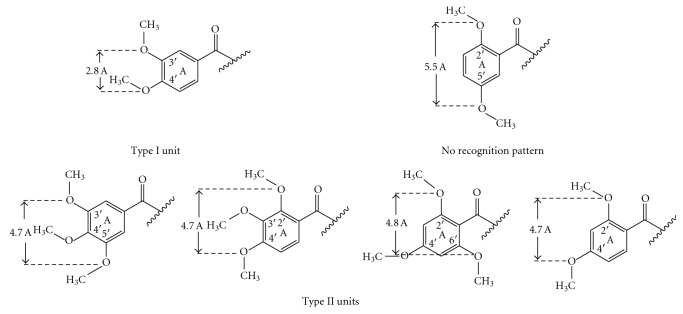
Recognition patterns of chalcones **1**–**18** required for the interaction with P-gp.

Methoxy groups are the most widespread structural elements in P-gp modulators [23]. They possess potent H-bond acceptor capacity and substantial lipophilicity, both in favor of the modulator properties. The spatial arrangement of the methoxy groups in P-gp inhibitors is of great importance because they can form recognition patterns necessary for the activity [7, 23]. Such patterns were defined by Seelig as type I (two electron donor groups with a spatial separation of 2.5 ± 0.3 Å) and type II (two electron donor groups with a spatial separation of 4.6 ± 0.6 Å or three electron donor groups with a spatial separation of the outer groups of 4.6 ± 0.6 Å). Chalcones in this study possess either type I (3′,4′-dimethoxy unit) or type II (all of the remaining units) recognition pattern (Figure 4). An exception was observed for 2′,5′-dimethoxylated ring A, which does not possess any of these types (Figure 4). This motif was only present in the least active compounds thus illustrating the necessity of a recognition model (Figure 1). Amongst the other dimethoxylated patterns, 2′,4′-dimethoxy unit was profitable for the activity of chalcones only when an *N*-containing basic (dimethylamino) group was present in ring B, which was in agreement with findings of Liu et al. [7]. While 3′,4′-dimethoxy unit was favourable only when nonbasic group (chlorine) was introduced in ring B. The corresponding chalcone **10** was the most active compound in this study, being 2-fold stronger P-gp inhibitor than the lead compound **1**. The most successful combination of three methoxy groups in ring A was that of the 3′,4′,5′-trimethoxy unit, followed by 2′,4′,6′-trimethoxy unit. However, effective spatial arrangement of methoxy groups in the ring A could not be defined without considering the type of the *p*-substituent in ring B.

Overall, the MDR reversal activity of chalcones decreased with decreasing the hydrophobicity of the *p*-substituent in ring B, as follows: 4-chloro > 4-dimethylamino > 4-methoxy group (Figure 1). This result is in favor of the hypothesis that the *p*-substituent of chalcones should be more hydrophobic to probably strongly bind to the steroid-interacting region of the P-gp's NBD2 [5]. Such tendency is also valid for the other types of flavonoids [24]. Each *p*-substituent in ring B required a specific methoxylation pattern in the ring A (Figure 4). Thus, presence of a *p*-chloro group was advantageous for the activity of chalcones with 3′,4′,5′-trimethoxy, and 2′,4′,6′-trimethoxy units, (having a type II recognition model) and provided the most active compound **10** having 3′,4′-dimethoxy unit (type I recognition model). The presence of *p*-dimethylamino group was of similar value for the activity of all chalcones with type II recognition pattern (3′,4′,5′-trimethoxy, 2′,3′,4′-trimethoxy, 2′,4′,6′-trimethoxy and 2′,4′-dimethoxy units) and it was not favourable for the activity of the chalcones with either type I or not having any type of the recognition model (3′,4′-dimethoxy- and 2′,5′-dimethoxy units). Meanwhile, all the chalcones with *p*-methoxy group were the least active compounds regardless of their methoxylation pattern in the ring A (Figure 1).

### 3.5. Antiproliferative Effects of Chalcones **1–18** on L 5178 Y Cells

Many cancer-related studies on chalcones have demonstrated the positive influence of 3′,4′,5′-trimethoxylated ring A on cytotoxicity [25, 26]. In this study, the chalcones meeting this structural requirement were the most active inhibitors of the proliferation of human *MDR1* gene-transfected mouse lymphoma cells (L 5178 Y) (Table 4). Other effective methoxylation patterns were the 2′,3′,4′-trimethoxy- and 2′,5′-dimethoxy units. Although 2′,5′-dimethoxy motif was not advantageous for the MDR reversal activity, it seemed to be in favour of the antiproliferative activity of the resistant cells. Regarding the *p*-substituent in ring B, dimethylamino group was present in the molecules of the most active and of the most inactive compounds. This result emphasizes the importance of the methoxylated pattern in the ring A for the cytotoxic activity of chalcones.

## 4. Conclusions

Based on our results, the following important conclusions can be made for the P-gp modulatory activity of the chalcones having a methoxylated ring A and *p*-substituent in the ring B.

(1) The methoxylation pattern in the ring A should possess either a type I or a type II of the recognition model proposed by Seelig [23], but in all cases, the presence of 4′-methoxy group seems to be necessary.
(2) The *p*-substituent in ring B should be hydrophobic. This result is in favour of the hypothesis that the *p*-substituent of chalcones should be more hydrophobic to probably strongly bind to the steroid-interacting region of the P-gp's NBD2 [5].

In addition, 3′,4′,5′-trimethoxy-, 2′,3′,4′-trimethoxy-, and 2′,5′-dimethoxy units are favourable for the antiproliferative activity of chalcones on human *MDR1* gene-transfected mouse lymphoma cells, especially, when *p*-dimethylamino group was present in the ring B.

These results may be useful for the design of chalcone-like P-gp modulators and drugs able to treat resistant cancers.

## Figures and Tables

**Table 1:** Substitution pattern and calculated log *P* values of the chalcones studied for their MDR reversal activity.

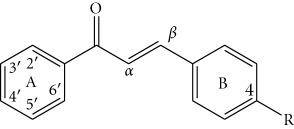				

Compound	Methoxylation pattern of ring A	R	log *P *	Reference^a^

**1**	3′,4′,5′-	–Cl	4.0	[8]
**2**	3′,4′,5′-	–N(CH_3_)_2_	3.5	[8]
**3**	3′,4′,5′-	–OCH_3_	3.3	[8]
**4**	2′,3′,4′-	–Cl	4.0	[9, 10]
**5**	2′,3′,4′-	–N(CH_3_)_2_	3.5	[10]
**6**	2′,3′,4′-	–OCH_3_	3.3	[10]
**7**	2′,4′,6′-	–Cl	4.0	[11]
**8**	2′,4′,6′-	–N(CH_3_)_2_	3.5	[11]
**9**	2′,4′,6′-	–OCH_3_	3.3	[11]
**10**	3′,4′-	–Cl	4.2	—
**11**	3′,4′-	–N(CH_3_)_2_	3.7	[12]
**12**	3′,4′-	–OCH_3_	3.4	[13]
**13**	2′,4′-	–Cl	4.2	[9]
**14**	2′,4′-	–N(CH_3_)_2_	3.7	[12, 14]
**15**	2′,4′-	–OCH_3_	3.4	[14]
**16**	2′,5′-	–Cl	4.2	—
**17**	2′,5′-	–N(CH_3_)_2_	3.7	[12]
**18**	2′,5′-	–OCH_3_	3.4	[15]

^a^Chalcones reported by other sources.

**Table 2:** Reversal of MDR by chalcones **4–18** on mouse lymphoma cell line L 5178 T—dose-response experiments.

Compound	Concentration [*μ*g/mL]	Fluorescence activity (ratio)
Verapamil	5.2 *μ*M	4.94
**1**	4	40.37^a^
	40	26.63^a^
**2**	4	5.54^a^
	40	33.16^a^
**3**	4	2.24^a^
	40	65.54^a^
**4**	4	2.93
	40	36.40
**5**	4	10.04
	40	49.28
**6**	4	2.86
	40	34.92
**7**	4	9.84
	40	29.11
**8**	4	9.68
	40	22.35
**9**	4	3.57
	40	12.26
**10**	4	41.65
	40	48.37
**11**	4	5.04
	40	83.80
**12**	4	2.84
	40	47.14
**13**	4	2.59
	40	39.93
**14**	4	9.81
	40	22.28
**15**	4	1.73
	40	37.85
**16**	4	1.54
	40	16.64
**17**	4	1.81
	40	16.47
**18**	4	2.48
	40	25.40
DMSO control	20 *μ*L	1.23

^a^The FAR values of chalcones **1**, **2,** and **3** were determined previously [8].

**Table 3:** 

FIC_A_ = ID_50A in combination_/ID_50A alone_	FIX = FIC_A_ + FIC_B_
FIC_B_ = ID_50B in combination_/ID_50B alone_	FIX = 0.5–1, additive effect
ID = inhibitory dose	FIX < 0.5, synergism
FIC = fractional inhibitory concentration	FIX = 1 ÷ 2, indifferent effect
FIX = fractional inhibitory index	FIX > 2, antagonism

**Table 4:** Antiproliferative effects of chalcones **1–18** on L 5178 Y cells [*μ*g/mL].

	Methoxylation pattern of ring A					
*p*-Substituent in ring B	3′,4′,5′-^a^	2′,3′,4′-	2′,4′,6′-	2′,4′-	3′,4′-	2′,5′-

N(CH_3_)_2_	<0.19	0.42 ± 0.14	≫50	43.18 ± 1.48	>50	0.47 ± 0.01
Cl	0.14	2.16 ± 0.00	5.19 ± 0.14	4.98 ± 0.33	2.17 ± 0.31	4.95 ± 0.12
OCH_3_	<0.19	2.23 ± 0.28	22.75 ± 3.90	42.58 ± 18.03	5.06 ± 0.89	1.74 ± 0.07

^a^The antiproliferative activity of chalcones having 3′,4′,5′-trimethoxylated pattern was determined previously [8].
